# Free-floating and spinning thrombus of the basilar artery: A case report

**DOI:** 10.1097/MD.0000000000025696

**Published:** 2021-05-28

**Authors:** Keon-Yeup Kim, Sang-Hun Lee

**Affiliations:** Department of Neurology, Korea University Ansan Hospital, Korea University College of Medicine, Ansan, Republic of Korea.

**Keywords:** basilar artery, cerebral ischemia, free-floating thrombus, spinning thrombus

## Abstract

**Rationale:**

Free-floating thrombi in the intracranial artery are rare. We report a case of a free-floating and spinning thrombus caused by turbulent flow distal to the basilar artery stenosis. We compare thrombus changes in a series of images according to time and describe the approach to treatment and thrombosis resolution.</abstract>

**Patient concerns:**

A 55-year-old man presented to the emergency department on March 21, 2020, with left-sided weakness, bilateral limb ataxia, and a one-day history of dysarthria. Brain magnetic resonance imaging showed multifocal infarctions in the pons and cerebellum with severe basilar stenosis.

**Diagnoses:**

Digital subtraction angiography showed severe focal stenosis. A relatively large oval-shaped mobile thrombus was observed spinning due to turbulent flow at the distal portion of the stenosis.

**Interventions:**

We administered a combination antithrombotic regimen of warfarin and clopidogrel for 50 days.

**Outcomes:**

No thrombus was observed on the third follow-up digital subtraction angiography.

**Lessons:**

No previous study has directly observed a mobile thrombus in the intracranial artery using digital subtraction angiography. We used a combination antithrombotic strategy, which was effective after long-term, rather than short-term, use.

## 1 Introduction

A free-floating thrombus has an incidence of 0.05% to 0.5%.^[1,2]^ and may involve several overlapping disorders including intraluminal thrombi, embolic thrombi, plaque thrombi, and mobile thrombi due to underlying lesions—including carotid aneurysms, small dissections, and atheromatous plaques.^[3]^ Hemodynamics affect floating thrombus formation, which may be accelerated in regions of high or recirculating flow and increased turbulence. However, the natural history and etiology of free-floating thrombi in the cerebral circulation are unknown.^[2]^ We report a case of a free-floating and spinning thrombus caused by a turbulent flow distal to the basilar artery stenosis. We compared several imaging techniques at the time and described a treatment approach to resolve the thrombosis. The patient provided informed consent for the publication of this case.

## 2 Case presentation

**Figure 1:**
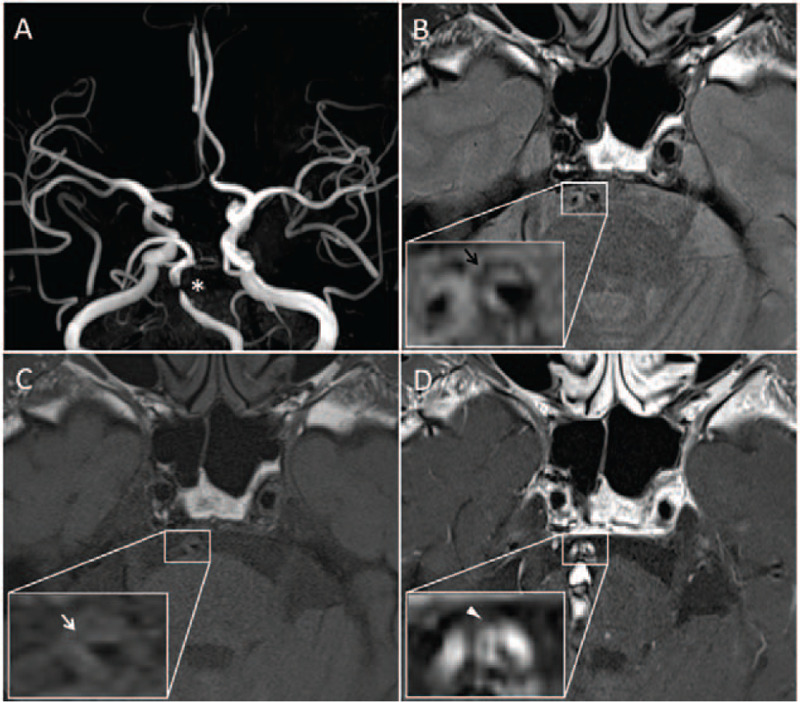
High-resolution magnetic resonance imaging (HRMRI) of our patient. (A) Time-of-flight MR angiography shows severe stenosis in the basilar artery (^∗^) (B) Proton density sequence shows eccentric wall thickening and atherosclerotic plaques (black arrow). (C) T1-weighted image of HRMRI shows eccentric atherosclerotic plaques without intraplaque hemorrhage (white arrow). (D) Contrast-enhanced T1-weighted image of HRMRI shows strong enhancement in eccentric atherosclerotic plaques (white arrowhead). HRMRI = high-resolution MRI, MRI = magnetic resonance imaging.

A 55-year-old male presented to the emergency department on March 21, 2020, with left-sided weakness, bilateral limb ataxia, and a one-day history of dysarthria. He had a history of hypertension; otherwise, he had no significant past medical history or family history of cerebrovascular disease or risk factors for atherosclerotic stroke. These symptoms developed a day before presentation. At the time of the visit, he had a blood pressure of 180/130 mm Hg, a pulse of 89 beats per minute, a body temperature of 36.4°C, and a respiratory rate of 16 breaths per minute. His neurological symptom score on the National Institutes of Health Stroke Scale was 4. Cardiac examinations including transthoracic echocardiography and 24-hr Holter monitoring were normal. Brain magnetic resonance imaging (MRI) was performed to confirm cerebral ischemia. Diffusion-weighted imaging showed multifocal infarctions in the pons and cerebellum with severe basilar stenosis. High-resolution MRI was performed to accurately assess the inner wall of the cerebral artery and to evaluate the etiology of the severe focal stenosis. Our institution's high-resolution MRI protocol involved 3D proton-density imaging with turbo spin-echo sequences using the following parameters: TR/TE=2,450/37 ms, FOV= 100 × 100 mm, and matrix size= 195 × 256. T1-weighted imaging and T1-enhanced imaging with turbo spin-echo sequences were performed using the following parameters: TR/TE= 670/9.1 ms, FOV= 100 × 100 mm, and matrix size= 282 × 256. The HR-MRI images revealed atherosclerotic plaques within the region of severe basilar stenosis with lipid cores and fibrous components, but without intraplaque hemorrhage (Fig. 1).

**Figure 2:**
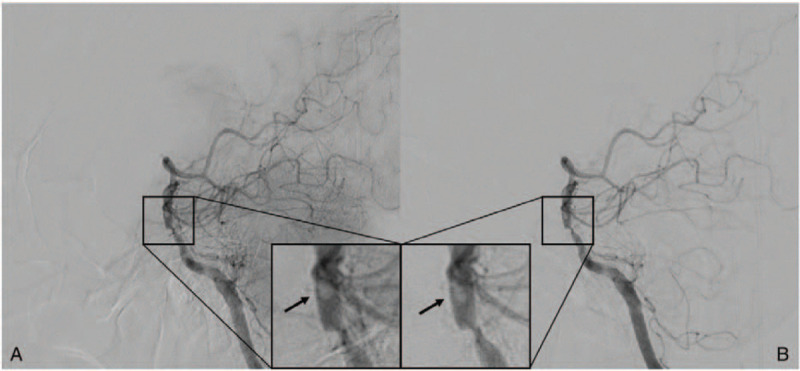
(A, B) First digital subtraction angiography (DSA) performed 3 days after the onset of symptoms shows a relatively large, oval-shaped, mobile thrombus (black arrow) spinning due to flow turbulence at the distal portion of the stenosis. DSA = digital subtraction angiography.

**Figure 3:**
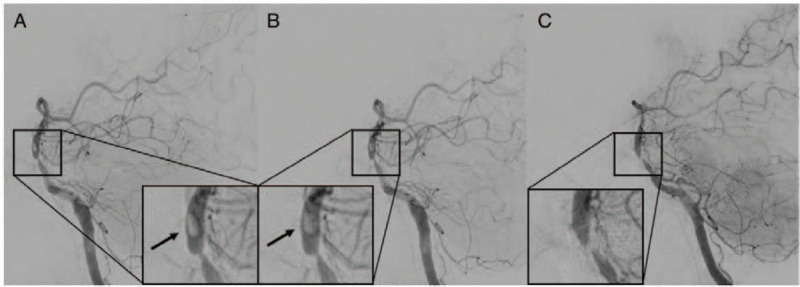
(A, B) Second digital subtraction angiography (DSA) performed 10 days after the onset of symptoms still shows the mobile and spinning thrombus (black arrow). (C) After administering anticoagulation therapy for 60 days, the mobile thrombus is not shown by the third DSA. DSA = digital subtraction angiography.

Digital subtraction angiography (DSA) was performed to evaluate the grade of arterial stenosis and perfusion on March 24, 2020. DSA revealed severe focal stenosis. A relatively large oval-shaped mobile thrombus was found spinning due to turbulent flow at the distal portion of the stenosis (Fig. 2 A, B). The risk of distal dislodging of the floating thrombus is very high when endovascular treatments such as angioplasty, using a balloon or stent insertion, is performed for the stenotic vessel. Consequently, we treated the patient with a combination antithrombotic regimen of warfarin and clopidogrel and strictly controlled the systolic pressure to < 130 mm Hg. DSA was repeated on March 31, 2020, and a spinning thrombus was still observed (Fig. 3 A, B). After administering combination antithrombotic therapy for 50 days, no thrombus was observed on the third follow-up DSA (Fig. 3 C).

## 3 Discussion

Our increased understanding of the etiology of ischemic stroke has directed attention toward intraluminal thrombi or intraluminal clots in the extra- and intracranial arteries.^[4]^ A catheter angiography study demonstrated that intraluminal thrombi account for 0.5% to 1.5%.^[4]^ This phenomenon is closely related to the direct induction of free-floating thrombi. Free-floating thrombi are rare, with an incidence of < 0.1%.^[1,3]^ No previous study has directly observed a mobile thrombus in the intracranial artery using DSA. We observed a free-floating and spinning thrombus was observed at the distal portion of the basilar stenosis.

The presence of an intraluminal or free-floating thrombus is a therapeutic challenge because of the associated risk of stroke. Also, empirical evidence to guide appropriate treatment strategies is lacking.^[5]^ Moreover, we do not know if neurointerventional treatments are safe for patients with intraluminal thrombi. These thrombi likely occur due to the distal dislodging of blood clots that occur during reperfusion.^[3]^ Complete dissolution of free-floating thrombi, without further neurological progression, occurred in 86% of patients treated with antithrombotic drugs.^[3]^ The most popular medical care plan was initial treatment with heparin, which switched from weeks to months to warfarin anticoagulant; some authors added antiplatelet drugs (aspirin or clopidogrel) to this regimen, while others only administered antiplatelet drugs.^[2]^ Although there are no randomized drug treatment trials, initial medical management with antithrombotic drugs (anticoagulants and antiplatelet drugs) is safe for most patients.^[3]^

We used a combination of antithrombotic regimens (warfarin and clopidogrel). A minimal change in the DSA shape was observed after 7 weeks, but it completely disappeared after 50 days. There were no additional neurological changes or deterioration between the treatments, and no additional ischemic changes were observed on follow-up MRI; therefore, our patient's thrombus resolved due to medical treatment, rather than distal migration.

In summary, we observed a floating thrombosis in our patient's intracranial artery. There was severe atherosclerotic stenosis with few neurointerventional options. We used a combination antithrombotic strategy, which was effective after long-term, rather than short-term, use.

## Author contributions

**Conceptualization**: Sang-Hun Lee

**Investigation**: Keon-Yeup Kim

**Methodology**: Sang-Hun Lee

**Resources**: Sang-Hun Lee

**Validation**: Keon-Yeup Kim

**Writing original draft**: Keon-Yeup Kim, Sang-Hun Lee

**Writing, review & editing**: Sang-Hun Lee
